# A Comprehensive Exploration of the Transcriptomic Landscape in Multiple Sclerosis: A Systematic Review

**DOI:** 10.3390/ijms24021448

**Published:** 2023-01-11

**Authors:** Luigi Chiricosta, Santino Blando, Simone D’Angiolini, Agnese Gugliandolo, Emanuela Mazzon

**Affiliations:** IRCCS Centro Neurolesi “Bonino-Pulejo”, Via Provinciale Palermo, Contrada Casazza, 98124 Messina, Italy

**Keywords:** multiple sclerosis, human analysis, non-coding RNAs, transcriptomic analysis, RNA-seq, spatial transcriptomics

## Abstract

Multiple Sclerosis (MS) is, to date, an incurable disease of the nervous system characterized by demyelination. Several genetic mutations are associated with the disease but they are not able to explain all the diagnosticated cases. Thus, it is suggested that altered gene expression may play a role in human pathologies. In this review, we explored the role of the transcriptomic profile in MS to investigate the main altered biological processes and pathways involved in the disease. Herein, we focused our attention on RNA-seq methods that in recent years are producing a huge amount of data rapidly replacing microarrays, both with bulk and single-cells. The studies evidenced that different MS stages have specific molecular signatures and non-coding RNAs may play a key role in the disease. Sex-dependence was observed before and after treatments used to alleviate symptomatology activating different biological processes in a drug-dependent manner. New pathways, such as neddylation, were found deregulated in MS and inflammation was linked to neuron degeneration areas through spatial transcriptomics. It is evident that the use of RNA-seq in the study of complex pathologies, such as MS, is a valid strategy to shed light on new involved mechanisms.

## 1. Introduction

Multiple sclerosis (MS) is a chronic neurodegenerative disease of the central nervous system (CNS), of which the accumulation of demyelinating lesions both in white and grey matter in the brain and spinal cord is the marker [1]. The challenge with MS is the early diagnosis of the pathology for the high percentage of conversion from clinically isolated syndrome (CIS). This syndrome is characterized by inflammatory demyelination even if the MS criteria are not entirely fulfilled. However, not all patients with CIS convert to MS [2]. The majority of patients show a relapsing remitting MS (RRMS) form, where reversible episodes of neurological deficits, indicated as relapses, characterize the initial disease stage. After 10–15 years, the majority of patients show a slow progression of the disease with permanent neurological deficits and a clinical disability that is known as secondary progressive MS (SPMS). Only a minority of patients are affected by primary progressive MS (PPMS), which shows a progressive disease course from the onset [3]. Another difference between the MS forms is related to the age of onset. Indeed, RRMS presents a precocious onset normally between 20 and 35 years of age. Conversely, PPMS onset appears close to 40 years of age [4]. It is estimated that about three million people are affected by MS, with a difference between sex because females are more affected than males [5]. Multiple sclerosis presents an autoimmune mechanism where the target is represented by myelin antigens. Both CD4+ and CD8+ T lymphocytes play a role in the pathological mechanism [4]. In MS, pathogenic T helper (Th) 17, Th1 and CD8+ autoreactive T cells are directed against myelin components. Moreover, in the demyelinated lesions, local microglia and macrophages are also activated [6]. Additionally, B cells play a role in the immunopathogenesis of MS. In the earlier stages of the disease, CD20+ B cells are the most observed, while in the progressive phase plasma-blasts and plasma cells are mainly involved [4]. B cell involvement is not only related to antibody production, but also to the antigen presentation to T cells and myeloid cell function modulation due to cytokine production [7].

Nowadays, the aim of the available therapeutic approaches is treating acute attacks and improving symptoms. Disease-modifying therapies, such as fingolimod and interferon (IFN) -β (IFN-β), are able to modulate the immune system, leading to a reduction of inflammation and of the rate of relapses. Nevertheless, even if they can stabilize or delay disease, only in some cases can they slightly improve disability [8].

Multiple sclerosis is a heterogeneous, multifactorial, immune-mediated disease that is caused by complex gene–environment interactions [9]. Genome-wide association studies found about 110 genetic variants associated with an increase in the risk of developing MS. Most of these genes are involved in immune mechanisms. Interestingly, some of these genes are shared with other autoimmune pathologies [10]. 

However, the susceptibility to MS is not adequately explained by the actually known genetic associations. Indeed, the known genetic variants associated cannot fully predict the susceptibility to MS. Moreover, nowadays the complex network of gene regulation is considered likely responsible for the complexity in humans [11]. In the case of MS, the complexity of the involved mechanisms suggests that a complex gene expression network may be involved in the disease. In light of this, it can be speculated that changes in gene expression profiles will provide valuable information regarding possible causes or consequences of the onset and progression of the disease. Indeed, it is more probable that changes in a group of genes, indicated as signatures, could potentially capture disease-specific alterations [12]. 

The term transcriptomics refers to the large-scale investigation of the comprehensive set of RNA transcripts produced by the genome. Transcriptomic studies provide both qualitative data, showing which transcripts are present, and quantitative data, showing the abundance of a transcript in a specific type of cell or tissue in a specific moment. 

The improvements made in omics science have allowed the production of huge amounts of data derived from different cellular levels such as transcriptomes. Different technologies have been used in the last decades, such as microarrays. Nevertheless, microarrays are limited in the analysis of transcriptomes because they are probe-specific, cannot identify new transcripts and it is not easy to compare the levels of expression of different microarrays with each other [13]. Recently, the RNA-seq analysis performed by high-throughput sequencing (HTS) experiments by Next-Generation Sequencing (NGS) technologies has increased the accuracy of data and made it possible to perform massive comparative transcriptomic studies. Bioinformatic analysis allows the analysis of transcriptomic profiles not only for the whole transcriptome but also for long non-coding RNAs (lncRNAs), small non-coding RNAs (sncRNAs), miRNAs and circular RNAs (circRNAs). Sometimes, alternative splicing and spatial transcriptomics as well as expression Quantitative Trait Loci (eQTL) and cis-splicing Quantitative Trait Loci (sQTL) have been used as alternative strategies to inspect transcriptome differences. Rarely, but interestingly, the transcriptomic level is also used to obtain genetic information relative to single nucleotide polymorphisms (SNPs). Indeed, unfortunately, it is not so frequent to have both genomic and transcriptomic profiles for a patient and, in this way, it is not so clear if transcriptome isolation plays a role in the disease [14]. Of note, it is not unusual to observe analysis conducted on the raw data of individuals retrieved from online databases. Indeed, this strategy allows the enlargement of the cohort and the production of more accurate results. Gene Expression Omnibus (GEO) [15], EBI-EMBL ArrayExpress [16] and DDBJ Sequence Read Archive [17] databases collect a huge amount of raw data from RNA-seq experiments. The included data can be obtained from the brain, cerebrospinal fluid (CSF), blood or peripheral blood mononuclear cells (PBMCs) and in different pathological or healthy conditions. Often, data not only include clinical but also registry records related to the uploaded samples so a matching for age and sex is possible. 

The comparative transcriptomic analysis of coding RNAs produces from hundreds to thousands of differentially expressed genes (DEGs) and bioinformatic analysis speeds up the studies through gene set enrichment analysis (GSEA). In particular, GSEA can be useful to understand which are the altered biological processes by inspecting Gene Ontology (GO). Gene Ontology is a database structured similarly to a dictionary and divided into the main categories “biological process”, “molecular function” and “cellular component” [18]. Usually, the terms derived from the biological process category are the most used to understand the biological changes in the MS condition or after a specific treatment. Additionally, GSEA can be used to study the pathways that contain the most DEGs. In detail, the Kyoto Encyclopedia of Genes and Genomes (KEGG) is the most-used database of the pathway because it contains manually curated molecular cascades of genes involved in metabolism, genetic information processing, environmental information processing, cellular processes, organismal systems, human diseases and drug development [19]. 

In this review, we summarize the studies that investigated through transcriptomic analysis the intricate gene expression network in MS. In particular, we considered the studies published in the last 10 years that evaluated the role of the transcriptomic profile obtained through bulk- or single-cell analysis by the RNA-seq method in MS. In this line, we focused our attention on the biological processes and pathways mainly altered in the disease.

## 2. Methods

In order to select the manuscripts, we proceeded searching on PubMed for “((multiple sclerosis(Title)) AND ((RNA seq) OR (transcriptomic)) NOT (microarray) NOT (single cell)) AND (“2012”(Date—Publication): “3000”(Date—Publication)) NOT (Review(Publication Type))”. On Web of Science, we searched for “TI = (multiple sclerosis) AND (TS = (transcriptomics) OR TS = (RNA-seq)) NOT TS = (microarray) NOT TS = (single cell)” excluding manuscripts published before 2012 using filters. The research was conducted on 19th September 2022.

Then, we performed a search for single-cell studies on PubMed using “((multiple sclerosis(Title)) AND (single cell RNA seq) NOT (microarray)) AND (“2012”(Date—Publication): “3000”(Date—Publication)) NOT (Review(Publication Type))” and on Web of Science for “TI = (multiple sclerosis) AND (TS = (single cell RNA seq)) NOT TS = (microarray) excluding manuscripts published before 2012 using filters. The research was conducted on 20th December 2022. 

In this line, all the publications from 2012 to 2022 were taken into consideration for this work. Then, articles that included RNA-seq data related to human samples were investigated.

## 3. Results and Discussion

**Figure 1:**
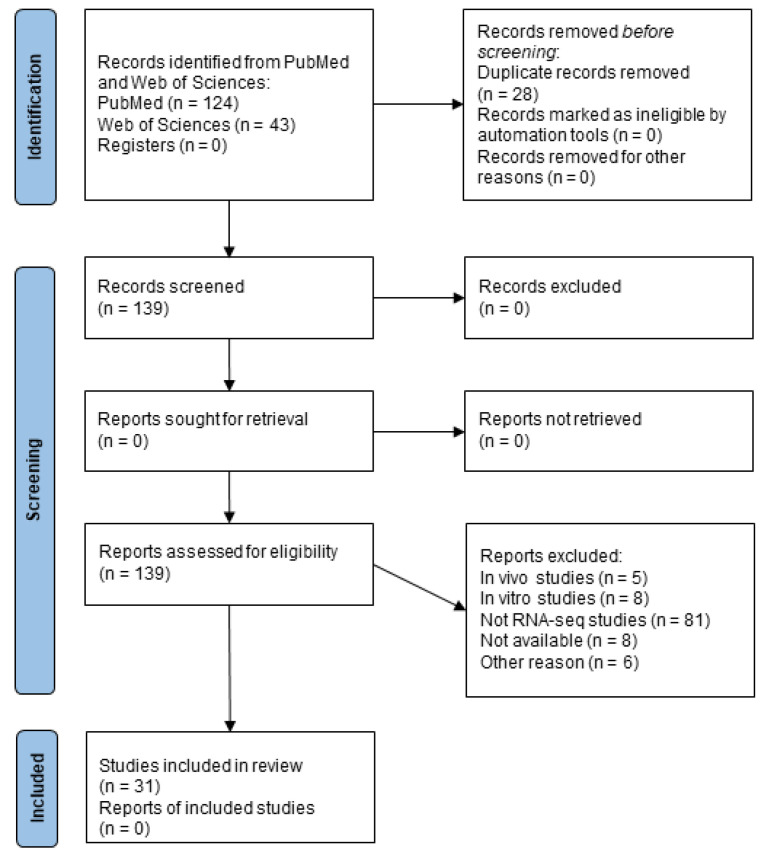
Prisma flow diagram of article selection for bulk studies.

**Figure 2:**
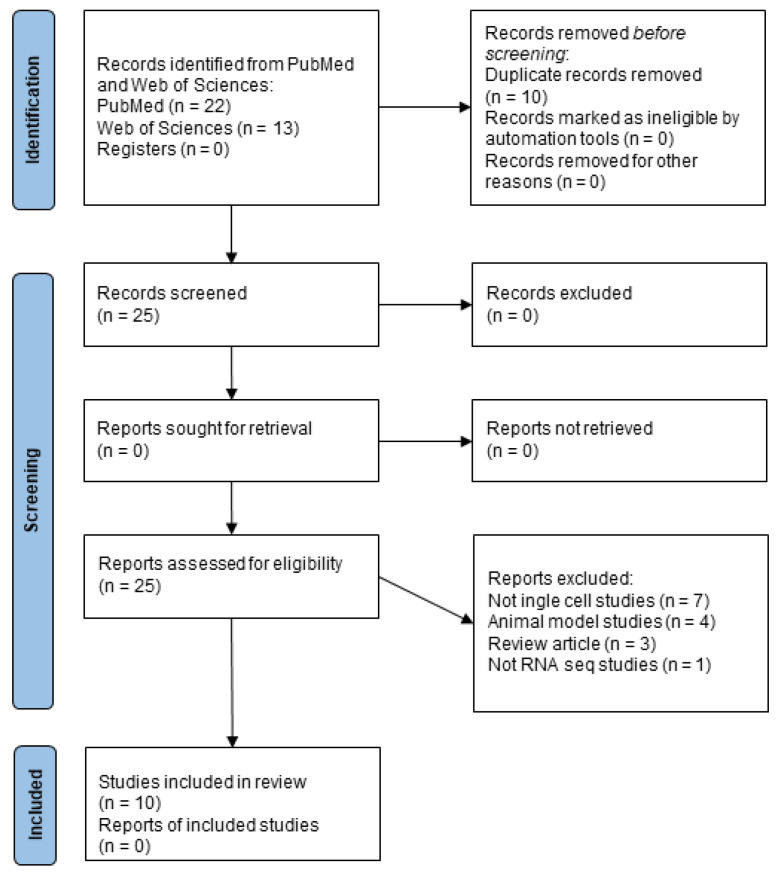
Prisma flow diagram of article selection for single-cell studies.

According to the research criteria reported in the Methods section, 31 papers were found regarding RNA-seq data related to human samples (Figure 1) and 10 articles for single-cell studies (Figure 2).

### 3.1. Human Sample Studies Exploring Transcriptome in MS 

**Figure 3:**
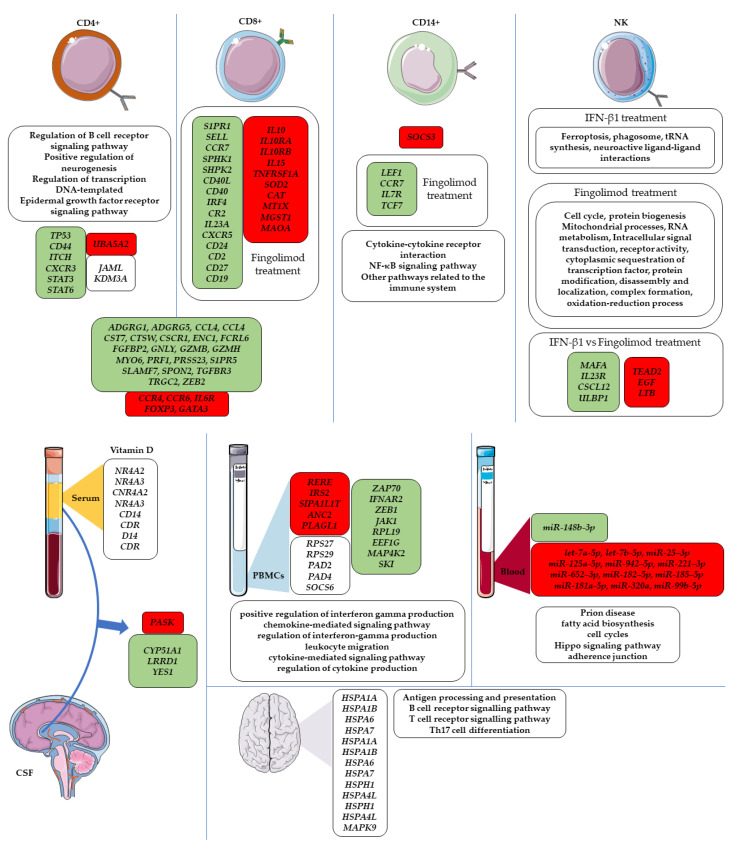
Summing up of the main conclusions of the studies of the review. In red are represented DEGs observed upregulated in the studies, while the green boxes represent downregulated ones.

Most of the cohort of collected patients related to RRMS, which is the most common form of the disease. Also, in some studies patients affected by PPMS and SPMS were included. The size of the cohorts was very different among the studies, ranging from a few units, in some studies, to hundreds in other. Mostly, this was related to the aim of the performed analysis along with the type of tissue that was taken under consideration. Indeed, even if the analysis could produce more accurate results, tissues retrieved through invasive methods are more difficult to obtain. Most of the studies focused on the comparative level of the expression of transcripts in MS against either a healthy state or a negative diagnosis for a neurodegenerative condition. In some cases, the studies inspected the transcriptomic profile of MS before and after a specific treatment. Finally, both bulk and single-cell studies were included. The main conclusions are summarized in Figure 3.

#### 3.1.1. Peripheral Blood Mononuclear Cells

Most of the studies on MS were conducted on PBMCs. Peripheral blood is quite easy to obtain, and PBMCs such as T cells, B cells and NK cells are known to be involved in the triggering or in the progression of MS. Thus, inspecting the changes in the transcriptomic profile of PBMCs in MS patients and in individuals that have never received a diagnosis for this disease is an optimal way to identify possible altered networks. In addition, transcriptomic analysis can be helpful to identify alteration in gene expression after specific treatments that hit the immune response [20]. Transcriptomic analysis gives us the possibility to inspect simultaneously the level of expression of the whole transcriptomic profile of a cell. In this sense, the transcriptome obtained from total RNA can reveal the landscape of the disease.

Luiz H. Nali et al. inspected the whole transcriptome of PBMCs collected from three patients with RRMS and six SPMS patients. The MS patients were compared against five healthy controls (HC), matched for age and gender. The comparison between the two MS populations showed different patterns of gene expression. In particular, the authors observed that the inflammatory profile was clearly activated in RRMS patients while in SPMS patients a decrease in pathways related to neuronal repair was observed. However, there were some overlapping patterns of genes. Indeed, *RERE*, *IRS2*, *SIPA1L1*, *TANC2* and *PLAGL1* are commonly upregulated in the two forms of MS. Conversely, *RPS27* and *RPS29* were deregulated in both of them. In addition, they observed an interesting behavior for *PAD2* and *PAD4* that resulted in upregulation in RRMS while the same genes were downregulated in SPMS, probably due to the degenerative profile and remyelination failure [21]. In the previous study, the whole transcriptome was made on PBMCs whereas Kicheol Kim et al. performed a study on fluorescence-activated cell sorting-sorted specific immune cell populations, CD4+ and CD8+ T cells and CD14+ monocytes. The cohort was composed of 106 patients affected by MS and 22 HC. Comparing the two groups, most DEGs were observed in CD4+ (479 genes) and in monocytes (435 genes) but only 54 DEGs were identified in CD8+ T cells. Among the MS subtypes, no DEGs were found. The highest fraction of DEGs identified in CD8+ T cells were observed downregulated in MS. The upregulation of *SOCS3* called attention to CD14+ monocytes. This gene is linked to the inflammatory state and was observed to match the upregulation of *IL1B* and *CSF2RB*. The authors focused attention on the *NAE1* gene. In CD4+, *NAE1* was one of the most upregulated and it is included in the main protein–protein interaction network. It is involved in the activation of the neddylation pathway. To explore the neddylation pathway, the authors inhibited the NEDD8 activating enzyme, of which *NAE1* is an important subunit in experimental autoimmune encephalomyelitis (EAE) mice. The inhibition reduced the severity of the disease. The results were confirmed by a reduction in demyelination and inflammatory cell infiltration [22]. Then, transcriptomic analysis was useful to highlight neddylation as a new therapeutic target for MS.

Zahara Salehi et al. studied the transcriptomic profile of CD4+ T cells obtained from the PBMC of four patients affected by RRMS during the relapse phase and compared it with four HC. The authors conducted an in silico GSEA. The enriched identified pathways were linked to the immune system (“regulation of B cell receptor signaling pathway”), to brain morphogenesis (“positive regulation of neurogenesis”) or with general processes (the “regulation of transcription, DNA-templated”, “protein phosphorylation” and the “epidermal growth factor receptor signaling pathway”). The key DEGs identified were *JAML*, involved in class I MHC-mediated antigen processing and presentation, and *KDM3A,* which participates in hormone-dependent transcriptional activation. Additionally, KEGG pathways associated with the immune system and viral infections were identified as enriched. The interaction network highlighted the upregulated *UBA52* and the downregulated *TP53* genes as hubs. Moreover, the authors associated the DEGs identified in the analysis with the T cell markers, observing a downregulation of *CD44*, *ITCH*, *CXCR3*, *STAT3* and *STAT6* during the relapse phase [23]. This approach can help to find novel genes and pathways involved in MS pathogenesis. In another study, Sunjay Jude Fernandes et al. associated the transcriptome with epigenomic analysis in a population of CD4+ and CD8+ T cells from patients with RRMS and SPMS in comparison with healthy subjects. In detail, the cohort was composed of 12 healthy subjects for CD4+ and 15 for CD8+ T cells, 12 RRMS patients for CD4+ and 11 for CD8+ T cells, 10 SPMS patients for CD4+ and 8 SPMS for CD8+ T cells. Considering the limited sample size, a non-parametric combination procedure was performed for the data integration to get stronger statistical assumptions. After trying to gather the cohort through Principal Component Analysis, no clustering was identified for any of the two T cell populations. Also, no gene was identified as differentially expressed in CD4+ or in CD8+ cells in the passage from RRMS to the SPMS condition. Nevertheless, from healthy to RRMS, 34 DEGs were identified in CD4+ and 14 DEGs in CD8+ T cells. The enriched analysis made on the highlighted DEGs showed an association of DEGs with the translation process and, curiously, the first ten biological and molecular pathways are the same for CD4+ and CD8+ T cells. Among the 149 DEGs identified in the analysis, their deregulation was clustered in five categories. Two of them mainly contain genes involved in mRNA processing and translation and were associated with modification into RRMS and SPMS conditions. Two other categories collected *BCL10*, *HLA-G*, *IGJ*, *SOX4*, *IFIT2*, *IRGM*, *F2RL1*, *STAMBP*, *GIMAP4* known to be involved in immune functions, *TNRC6B* and *TDRKH*, which participate in RNA regulation, and *GMFB* and *NEFL*, which have specific functions in neurons. Similarly, the authors conducted the experiment with a focus on methylation patterns observing the hypermethylation of *SH3YL1*, which matches with its downregulation in RNA-seq analysis [24].

The comparative transcriptomic analysis between MS patients and controls highlights key differences specific to the disease. Nevertheless, the whole transcriptome can also evaluate patterns of deregulated genes as a consequence of a treatment, or in responders compared to non-responders. Hanna Charbit et al. inspected the deregulation of 526 DEGs related to the immune system that changed their behavior after IFN-β treatment in 77 RRMS patients. The authors used the PBMCs of 30 patients (21 women and 9 men) and performed a comparative transcriptomic analysis among the 16 patients that improved their condition after treatment against the 14 patients for whom no change was observed. The modification in the expression followed a sex issue; indeed, *ZAP70*, *IFNAR2*, *ZEB1*, *JAK1*, *RPL19*, *EEF1G*, *MAP4K2* and *SKI* were deregulated in men but not in women. Then, the authors validated the expression of their results on 47 external patients, 28 women and 19 men, that were confirmed for *JAK1*, *IFNAR2* and *SKI*. Considering the expression of the eight genes reported, it is possible to predict the therapy response but in a sex-specific manner [25]. Sferruzza, F. et al. inspected the effects of fingolimod in the CD14+ monocytes of RRMS patients before and 6 months after treatment. After comparative transcriptomic analysis, 60 DEGs were observed downregulated, among which *LEF1*, *CCR7*, *IL7R* and *TCF7* were the most representative. The GSEA of DEGs enriched “Cytokine-cytokine receptor interaction”, the “NF-κB signaling pathway” and other pathways related to the immune system. Additionally, the “Wnt signaling pathway”, “Chemokine signaling pathway” and “Rap1 signaling pathway” were associated with the treatment. A network analysis between DEGs and their possible interactors in monocytes highlighted 4751 nodes and, after ribosomal gene removal and module inspection, the remaining 444 nodes, such as *IL7R*, *CTS* and *TNFAIP3*, were associated with the migration and activation of the white blood cells. Thus, the authors observed modulation in the activity of myeloid cells due to the drug possibly mediated by the Wnt pathway [26]. Nazire Pinar Acar et al. focused their attention on the treatment given to 91 RRMS patients and the changes in the transcriptomic profile of NK cells. In detail, 55 patients were treated with fingolimod, among which 15 patients were treated for a month, 16 patients for 3–6 months and 24 patients for more than a year. Additionally, 36 patients were treated with IFN-ꞵ1 for more than one year. The comparison was also made against 9 patients with a new diagnosis and against a control group of 26 individuals. All the samples were matched for age and sex against the first group treated with fingolimod. The PBMCs were obtained during the phase of remission for MS patients. They used fluorescence-activated cell sorting to study the cell population, finding a lower number of CD56^bright^ after treatment with fingolimod while no significant changes were found in CD56^dim^ in comparison with IFN-ꞵ1. Even if both treatments produced improvements in the condition of patients, the two compounds altered the transcriptomic profiles in different ways. A first comparison of NK between healthy patients and patients with a new diagnosis revealed 1988 DEGs while against the IFN-ꞵ1 group 2405 DEGs were observed. Most of the DEGs were upregulated in the MS groups. On the other hand, against the fingolimod group 1889 DEGs were observed, mostly downregulated. An interesting result was shown by the comparison of the two treatments, where 3249 genes resulted in downregulation, most of them upregulated in fingolimod. *MAFA*, *IL23R*, *CXCL12* and *ULBP1* were the most downregulated DEGs while *TEAD2*, *EGF* and *LTB* were the most upregulated ones. Also, the NK cells in patients treated with fingolimod were observed to be similar to healthy ones. The analysis of the pathways in which DEGs fell found they were associated with neuroactive ligand–receptor interactions, ferroptosis, phagosomes and tRNA synthesis after treatment with IFN-ꞵ1. The fingolimod group showed a downregulation of genes belonging to the cell cycle, protein biogenesis, mitochondrial processes, RNA metabolism and intracellular signal transduction pathways while an upregulation of ones in the regulation of receptor activity, the cytoplasmic sequestration of transcription factors, protein modification, disassembly and localization, complex formation and the oxidation-reduction processes. The results suggested that for patients that have not achieved any benefit from the first-line of disease-modifying drugs, such as IFN-β1, fingolimod can be a really interesting choice [27].

Along with fingolimod and IFN-ꞵ1, vitamin D is thought to play a role in MS. Indeed, high levels of vitamin D in serum are known to reduce MS disability over time and low latitude is associated with high levels of vitamin D, so the relationship between latitude connected to sun exposure and MS severity score was studied. All the data related to serum were retrieved from NationMS (n = 908) and BIONAT (n = 808). For RNA-seq, the PBMCs of five patients from a 2014 pilot study were used. Even if high levels of vitamin D are an important protection factor for MS, exceptions were observed on the island of Sardinia, in Italy, where high exposure to UVR and the prevalence of MS were both present. A lower tendency and worsening of the disease were observed for the patients of the NationMS cohort with a higher level of vitamin D. The vitamin D results strongly associated with latitude, so this element is directly related to MS severity and worsening. Only in patients not previously treated with IFN-β were the effects of latitude observed. This is related to the modulation of IFN-β towards vitamin D synthesis. Transcriptomic analysis sheds light on the expression of important genes associated with vitamin D activity such as *NR4A2*, *NR4A3*, *CD14* and *VDR*. Noteworthily, the characteristics of each patient, such as skin type and photosensitivity, need to be take into consideration because they play a critical role in the effect of the sun exposure that seems to be beneficials for MS patients [28].

Taking together all the available information of the disease, Irene Moreno-Torres et al. suggested the features that could be used by a model to predict how MS patients respond to treatment by inspecting a cohort of 50 RRMS patients and 10 controls with ages ranging from 18 to 55 matched in age and sex. Among the MS patients, only 10 of them were never treated with any drug. From the 10 selected patients, the PBMCs were obtained at time 0 and after 6 months of treatment with fingolimod. Transcriptomic analysis was used to obtain the expression of 48 subpopulations of lymphocytes. Scores of NEDA-3 (no evidence of disease activity) and NEDA-4 were measured 1 and 2 years after the beginning of fingolimod treatment for all the patients. This measurement allowed them to investigate associations with molecular and cellular characteristics. Controls and patients before treatments showed comparable levels of lymphocyte populations but LB1 cells. The treatment with fingolimod reduced the percentage of almost all the populations. When the treatment was not started, IFN-γ-producing CD4+ cells and effector memory T CD4+ correlated positively and plasmablasts with NK bright cells in turn negatively correlated with NK dim cells. At this step, NK bright, NK dim, plasmablasts along with IL-2 producing cells can be used as biomarkers following logistic regression analysis. After treatment, Helper T cells positively correlated with B cells (memory and regulatory), and negatively with naive B and NK cells. There, only naive CD8+ T cell populations were observed as possible biomarkers. The transcriptomic profile showed 7546 DEGs after treatment. Among the 3741 downregulated genes, *S1PR1*, *SELL*, *CCR7*, *SPHK1* and *SHPK2* were observed. The inflammation was reduced by the downregulation of *CD40L*, *CD40*, *IRF4*, *CR2*, *IL23A*, *CXCR5*, *CD24*, *CD2*, *CD27* and *CD19* and the upregulation of *IL10*, *IL10RA*, *IL10RB*, *IL15* and the *TNFRSF1A* receptor. Also, the upregulation of *SOD2*, *CAT*, *MT1X*, *MGST1* and *MAOA* was associated with reactive oxygen species presence. Almost a thousand genes were observed downregulated, more in responder than in no responder patients. The compound was able to hinder the apoptosis in the S1P pathway, reducing the expression of *AKT* and *NOS3* as well as T cell maturation through NFKB. Responder and non-responder groups mainly differ in processes related to cytokine secretion, the activation of the T cell response, the activation of regulatory mechanisms, immunoglobulin secretion, apoptosis, the processing of class I MHC, the innate immune response, the regulation of the Th1 response, immunoglobulin receptors and B lymphocyte response. Thus, the treatment revealed the change in the profiles of the lymphocytes acting particularly on transcription factors, moving from an anti-inflammatory to an antioxidant context [29].

Even if the whole transcriptome can provide the full landscape for the study of a specific condition, the focus on a particular subset, as on miRNAs, can also provide a huge amount of information with lower complexity of analysis. The work of Sanders et al. underlines, through the study of the miRNA expression profile in CD4+ T cells, that SPMS is a non-inflammatory mediated disease. The authors isolated PBMCs from the blood of SPMS patients. The initial cohort was composed of 12 SPMS patients and 12 HC. In addition, a replication cohort was composed of 12 SMPS patients and 10 HC. Some miRNAs highlighted in the work had an expression that contrasted the expression observed in RRMS patients. In SPMS patients, miR-155-5p, an miRNA with a proinflammatory role in MS, was found downregulated. The expression of different miRNAs analyzed in the work suggested that *SOCS6* is a novel gene that can play an interesting role in MS. This gene is highly conserved and shows a low expression in the brain and thymus tissue. It is also downregulated in pancreatic pathologies, colorectal and gastric cancers. *SOCS6* is a predictive target of the majority of miRNAs reported in the literature, and it is deregulated in the SPMS condition. This gene acts as a negative regulator in the activation of T cells but there is a small amount of information about it. To confirm *SOCS6* as a possible therapeutic target, more studies are required. The expression of *SOCS6* in SPMS is more evidence that CD4+ T cells play a diminished role in the pathologies that can be considered as a neurodegenerative stage and not a disease driven by inflammation [30]. Igor Selmaj et al. investigated the transcriptomic profile of the exosomes obtained by serum in 10 MS patients in the remission phase and 9 in the relapse phase in comparison to HC. Particular attention was paid to miRNAs and, in relapse, hsa-miR-122-5p, hsa-miR-196b-5p, hsa-miR-301a-3p and hsa-miR-532-5p were downregulated. The obtained results were then confirmed against an additional 33 MS patients in the relapse phase, 30 MS patients in the remission phase and 32 HC. Interestingly, the downregulation of the 4 miRNAs is in compliance with the magnetic resonance imaging obtained from the patients positive for Gd-enhancing lesions. Indeed, the lesions correlated with lower levels of miRNAs in serum. The authors inspected in vitro PBMCs and concluded that these cells might be the source of these miRNAs in the serum because of the correlation of their level of expression [31]. 

There are matter lesions present in the spine or brain in Radiologically Isolated Syndrome (RIS) along with MS. Nevertheless, RIS does not show any symptoms. Follow-up studies of patients with RIS have revealed that 34% of them develop MS over a 5-year period and 51.2% develop MS over a 10-year period [32]. For this reason, different studies are focused on finding the markers that can predict the conversion of RIS into MS. One crucial point in common with MS, along with other lesions, is the oligoclonal band biomarker. Kozin et al. aimed to answer this question using PBMCs isolated from eight RIS patients, using RNA-seq. The PBMCs from eight HC were also obtained. All RIS patients had oligoclonal bands and lesions. Among the significantly altered pathways, “positive regulation of interferon gamma production”, “chemokine-mediated signaling pathway”, “regulation of interferon-gamma production”, leukocyte migration”, “cytokine-mediated signaling pathway” and “regulation of cytokine production” are involved in immune response. These pathways are also involved in MS. The authors made a comparison taking advantage of Achiron et al., focused on the early stage of MS. They also chose the work of Achiron et al. for comparing the different DEGs because the study was conducted in PBMCs extracted from patients at early-stage MS. Kozin et al. identified *TNF*, *NR4A3*, *GIMAP4* and *PTGDR* as common DEGs. The RIS patients revealed a different PBMC transcriptomic profile compared to HC and the pathways involved in these changes involved the immune system. In this line, deep investigations could identify possible biomarkers able to predict the RIS to MS transition [33].

Transcriptomic analysis on PBMCs allowed the study of different lymphocyte populations in order to find alterations in DEGS and miRNA, differences among the MS subtypes and the study of profiles linked to drug treatments. The studies highlighted different interesting genes. *PAD2* and *PAD4* were upregulated in RRMS and downregulated in SPMS. In the CD14+ monocytes of the MS patients, *SOCS3* was upregulated and the neddylation pathway played a central role in demyelination and the inflammatory process. Another novel gene involved in the pathology is *SOCS6,* involved in cytokine signaling. Another important gene is *SH3YL1*, which was hyper methylated in PBMCs. Different studies focused on fingolimod and IFN-ꞵ1 treatment. Transcriptomic analysis showed different DEGs in the treatments. An important finding is that treatment with INF resulted in different DEGs in men and women. This difference highlights the importance of transcriptomic analysis for personalized treatments, with the aim of the creation of different drugs based on patients and the stages of the disease.

An overview the studies exploring transcriptomes in human PBMCs is reported in Table 1.

#### 3.1.2. Cerebrospinal Fluid

Since MS is an autoimmune disease, a key component to understand and treat the disease is the immune system. For this reason, many of the studies are focused on PBMCs because they are easily obtained from a blood sample even if the most interesting immune component is definitely the one in the nervous system. For these reasons, a way to make a diagnosis of MS is to visualize oligoclonal IgG bands in CSF on isoelectric focusing gels. However, the problem concerns the obtaining of samples at more time points. Transcriptomics in recent years is revolutionizing the understanding of diseases through immunoSEQ. The combination of transcriptomics with mass spectrometry clarified the understanding of oligoclonal IgG bands. The combination of these techniques allowed the transcripts of the heavy chain immunoglobulin gene (IGHV), which are known to be highly variable in the cells of the immune system, to be analyzed with extreme precision. The data of the transcripts of immunoglobulin are compared and integrated with the data of the mass spectrometry. Brändle et al. studied the oligoclonal bands (OCBs) using mass spectrometry and HTS. The main problem for the study of OCBs was the difficulty to identify a specific antibody because OCBs are hidden in a background of polyclonal antibodies. In this line, the authors used G affinity chromatography to select the IgG, after which they used two nonreducing 2D-Pages to obtain only the H2L2 complex. A key step was the copurification of the disulfide-linked IgG-H and -L chains from single OCB spots. This method yields intact H2L2 complexes. The spots containing the desired complex were then analyzed by mass spectrometry. The results were aligned to the specific Ig transcriptome obtained through the sequencing of the amplicons of the IgG genes. When mass spectrometry and NGS matched, they proceed to obtain recombinant OCB (rOCB) antibodies. They extracted CSF from one patient with neuroborreliosis and from 4 RRMS patients. It showed that the rOCB antibody created from a patient with neuroborreliosis binds Borrelia to verify the validity of the method. They produced six rOCBs of four RRMS patients and characterized three different target autoantigens. The three antigens bound ubiquitous intracellular proteins without brain tissue specificity. They proved that the B cell response in MS is heterogeneous and partly directed against intracellular autoantigens released during tissue destruction [34]. Also, N. Johansen et al. used mass spectrometry and HTS, but they focused on IgG-producing B cells in the CSF of 10 RRMS patients compared to 6 patients in a control group of other inflammatory neurological diseases. They collected CSF by lumbar puncture and CSF mononuclear cells were isolated. They also extracted PBMCs from blood. They used the HTS of transcribed IGHV genes and mass spectrometry. The sequencing of cDNA from the CSF was made using the immunoSEQ survey level assay. This assay was capable of identifying the entire spectrum of unique VDJ combinations. Mass spectrometry was performed on the IgG of the CSF. They found in both groups that the transcriptomes were dominated by a few B cell clones. In both groups, the B cells could mature on both sides of the blood–CSF barrier. All examined MS patients were closely related to IGH–VDJ transcripts on both sides of the blood–CSF barrier. A difference between the MS and the control was that the IgG-producing CSF B cells in MS patients used more frequent IGHV4 genes with a high number of replacement mutations [35]. A. Tomescu-Baciu et al. studied the intrathecal humoral immune response in two RRMS patients at two different time points. The time span between the sample collections was 18 months. The patients showed different disease duration and treatment: one was treated with natalizumab, the other with IFN-β1a. For the study, they isolated the PBMCs from blood and used the immunoSEQ and HTS of transcribed IGHV genes and the mass spectrometry of OCB IgG. The data showed that, despite the differences of the patients and the passage of time, there was a qualitative and quantitative persistence of oligoclonal IgG in the CSF of the two MS patients. Therefore, despite the exchange across the blood–CSF barrier of B cells during MS, there was a persistence of intrathecal oligoclonal B cells [36].

The studies on CSF highlighted the possibility to distinguish different B cell populations in MS patients, both in blood and CSF. It is known that blood and CSF are separated by BBB but cell, water and molecule exchange is allowed to some extent. Mass spectrometry, HTS and chromatography identified three antigens that are expressed in MS and, even though they are not specific to CSF, the technique may represent a turning point for other diseases. On the other hand, HTS and mass spectrometry revealed *IGHV4* as a highly specific gene for B cells belonging to CSF in MS patients and this can be explained by the finding of specific B cell clones that remain in the CSF of MS patients, despite the exchange allowed by BBB.

An overview the studies exploring transcriptomes in the human CSF is reported in Table 2.

#### 3.1.3. Blood

Different tissues can communicate with each other through the blood sending molecular signals. Peripheral blood collection is a less invasive way to study biomarkers and could be used to achieve early diagnosis and monitor the progression of neurological diseases [37]. 

In a study, particular attention was paid to the differences in the transcriptome profile of MS and controls based on the time of day. Among the seven recruited patients with RRMS, four were in remission phase, two in acute relapse phase and one was in remission with a recent relapse history. For each patient, the blood collection was repeated twice a day, at 2 pm (day) and 9 pm (night). In this way, it was possible to compare different phases of RRMS at different times of sampling. The results underlined some differences in gene expression in the relapse phase at night if compared with the remission and relapse phase during the day. The differences in expression were focused on genes related to immune response. In this line, the expression of genes in blood can indicate the state of the disease phase and the time of sampling can alter the result in a complex pathology such as MS [38].

Peripheral blood makes it possible to include genomic information in the transcriptomic levels through techniques of quantitative trait locus. 

Yijie He et al. used sQTL to map the splicing information due to genomic variants in a cohort of 51 MS patients and 91 HC. The analysis highlighted 5835 variants in 672 alternative splicing (AS) locations with a particular attention to the sites in which transcription starts. The results suggested that the intronic variants were more able to regulate events associated with AS and those related to retained intron (INT) AS were more influenced by genome variants. Also, GSEA was performed to find interaction profiles and showed a contribution to the modification of the phosphorylation phenomenon. [39].

Han et al. in their work analyzed simultaneously the lncRNA expression and the SNP genotype data starting from a cohort of 51 MS patients and 91 HC obtained from the GEO data set. The analysis was conducted using RNA-seq data and eQTL. The lncRNAs resulting as DEGs in the comparison between MS and HC were 2383. High numbers of lncRNAs resulting as DEGs are specifically expressed in the 13 brain regions. In addition, using the 3.2 billion reads obtained from the samples, it was possible to genotype a total of 600872 SNPs. In this line, 1054 highlighted cis-eQTL SNPs were identified as able to modulate the level of expression of 517 lncRNAs. For 17.6% of these lncRNAs the secondary structure of the transcripts is influenced by cis-eQTL SNPs. In addition, weighted gene coexpression network analysis (WGCNA) and GSEA demonstrated that the dysregulation in expression of lncRNAs caused by SNPs altered the antigen processing/presentation and MPAK signaling pathway in MS [40].

Beyond the longer length, lncRNAs differ from small RNAs, such as miRNAs, acting as silencers or droppers of the gene expression level after transcription.

Maria Liguori et al. in their study examined the transcriptomic profile of blood in 19 pediatric patients with RRMS against 20 healthy individuals of comparable age. The comparative transcriptomic analysis showed 49 deregulated miRNAs but the main differences were identified for let-7a-5p, let-7b-5p, miR-25–3p, miR-125a-5p, miR-942–5p, miR-221–3p, miR-652–3p, miR-182–5p, miR-185–5p, miR-181a-5p, miR-320a, miR-99b-5p, which were upregulated, and miR-148b-3p, which was downregulated in MS. The levels of these miRNAs were finally validated in the blood of the cohort through qRT-PCR. The GSEA conducted on the validated miRNAs identified Prion disease, fatty acid biosynthesis, cell cycles, the Hippo signaling pathway and adherence junction processes as enriched KEGG pathways. Additionally, 2215 upregulated and 2091 DEGs identified as mRNAs were observed. Among them, 68 downregulated DEGs were predicted as targets of deregulated miRNAs. *E2F2*, *DIP2A*, *GID4*, *GOLGA8A*, *KLHL14*, *CDC34*, *RPL35A*, *MICAL3* and *DNAJA4* were observed as targets of two or more of let-7a-5p, let-7b-5p, miR-181a-5p, miR-320a, miR-25–3p, miR-185–5p and miR-125a-5p miRNAs [41]. The event of RNA splicing can also produce a non-linear category of small RNAs, known as circRNAs, used by the organism to send biological signals. The cohort of Leire Iparraguirre et al. was composed by 120 patients affected by MS and 66 HC with a correspondence in age and sex among the groups. The samples were collected from whole blood. A first cohort for MS was made up of 30 MS patients (20 RRMS and 10 SPMS) along with 20 healthy controls (HC). A second cohort of 70 MS patients (62 RRMS and 8 SPMS) along with 46 HC were collected to validate the RNA-seq data with RT-qPCR. The expression obtained for circRNAs was studied in a third cohort of 20 RRMS patients. Even if a similar profile of circRNAs was found in the healthy and MS cohorts, in MS more than 95% was discovered upregulated in comparison to healthy subjects. Interestingly, the six circRNAs identified as altered and validated in the whole cohort in a sex dependent manner. Indeed, in this case, the six upregulated circRNAs were significantly observed in women but only three of them were identified in men [42].

Blood analysis allowed us to analyze multiple sclerosis in various aspects, in particular the various phases of the disease. The gene expression in MS in relapse was significantly changed at night compared with either relapse during the day or MS in remission. For example, the mRNA levels of *IL18R1*, *TPST1*, *TLR2* and *CXCR4* increased during the night. In addition, other work has shown that MS affects alternative splicing and these mRNAs can be the targets of deregulated miRNAs. The upregulation of six circRNAs in the blood suggested the increased incidence of the disease in females. Also, lncRNAs were studied using whole blood and this revealed that many of them expressed in cerebral and closer tissues carried SNPs responsible for the alteration of the secondary structure of lncRNAs.

An overview the studies exploring transcriptomes in the human blood is reported in Table 3.

#### 3.1.4. Brain

Most phenotypic changes in MS occur in the nervous system, where demyelination occurs. For this reason, when possible, the autopsy of MS patients for comparison with the brain tissue of patients who did not die from neurodegenerative conditions is the best way to observe differences. Nevertheless, retrieving a big cohort of this kind of samples is not easy. In this line, “MS Atlas” was realized by Tobias Frisch et al. It is the first interactive platform of web-based data analysis collecting the expression of lesions in MS. The database has collected the transcriptomic profiles of 98 human brains obtained from autopsy. In detail, the samples derived from 10 patients affected by PMS along with 5 controls that did not have a diagnosis of neurological conditions. At the time of death, the mean age of the patients was 52.4, while 56.4 for controls. The included lesions were related to 20 normal-appearing white matter samples, 17 active and 14 inactive, 17 chronic active and 6 remyelinating ones. The main aims of the authors were the discovery of biomarkers, gene panel comparisons and de novo network discovery [43]. Online databases allow us to collect huge amounts of data coming from different sources and perform quite accurate analysis. Our research group took advantage of the GEO repository to retrieve the transcriptomic profiles of the different brain areas of five patients who died with MS and healthy subjects. The in silico study was focused on the corpus callosum, hippocampus, internal capsule, optic chiasm, frontal and parietal cortex. Most of the DEGs were observed in optic chiasm and in corpus callosum while the lowest set of DEGs in cortex areas. The interesting deregulation of the proteins that are members of the heat shock protein family was highlighted in each of the six brain areas and all of them but one were upregulated in MS patients. *HSPA1A*, *HSPA1B*, *HSPA7*, *HSPA6*, *HSPH1*, *HSPA4L* were the always upregulated heat shock proteins while *HSPA2* was downregulated in the optic chiasm. As the authors underlined, a little upregulation of the heat shock proteins could be associated with a protective role whereas their extreme upregulation could exacerbate the disease, triggering the immune system. Inspecting the KEGG pathways “Antigen processing and presentation”, “B cell receptor signaling pathway”, “T cell receptor signaling pathway” or “Th17 cell differentiation” related to the immune system, all DEGs were upregulated except *MAPK9* in the internal capsule [44]. 

The use of databases makes it possible to increase the size of the cohort and perform predictions through artificial intelligence methods that require huge amounts of data. CIBERSORTx is a machine learning algorithm that was used by Sai Batchu to observe how M2 macrophages can lead to lesions. From a cohort of 10 MS patients in the progressive phase and 5 controls, he inspected 92 lesions related to chronic active, remyelinating, normal appearing white matter. The authors searched for the presence of M2 macrophages in white matter using a background dataset of 547 genes for 22 hematopoietic cells. The authors observed a higher abundance of M2 in the lesions of MS during the inactive phase [45]. 

Unfortunately, nowadays, not so many data about new RNA-seq strategies are included. Spatial transcriptomics was developed very recently and associates a type of cell of the histological section to a specific location taking advantage of the mRNA profile [46]. Max Kaufmann et al. created a new framework on which is based the development of drugs in association with MS. In a cohort of cortical brains from 13 SPMS and 5 controls from post mortem tissue, they inspected 37 tissue sections where 32 of them belonged to MS and 5 to controls, 1 from white matter and the other from gray matter. In grey matter, they firstly identified the profile of a neuron in both conditions and then observed a change in MS indicating neurodegeneration. Also, the spatial transcriptomics allowed them to find a not homogeneous kind of degeneration both in tissues and in cohorts. Then, genes with higher changes in expression were enriched for GO to inspect the progression of the disease. Biological processes associated with synapses such as synapse assembly, synaptic communication and plasticity were identified. Also, the regulation of neuronal projections and myelination were observed as well as immune cell activation and tissue remodeling. Through ulterior single-cell data, most of the genes observed deregulated were then mainly associated with a specific kind of cell. Nevertheless, the simultaneous deregulation of the same genes highlighted a cell type interconnection able to mediate the progression of the disease. The authors observed the lack of neuronal growth along with an inflammatory profile when the disease started [47]. 

The transcriptomic data obtained from the brain tissue of MS patients allowed the creation of an Atlas, which can help future scientists to speed up their research. The utility of online databases is reflected by the finding, in a dataset obtained from MS patients, of the extreme upregulation of heat shock protein family members. This phenomenon, associated with the upregulation of genes related to immune system pathways, suggests an exacerbation of the disease. The overactivation of the immune system can lead to inflammation, which was found associated with a deficit in neuronal growth.

An overview the studies exploring transcriptomes in the human brain is reported in Table 4.

#### 3.1.5. Multiple Tissue Comparisons

Taking into consideration the complex etiopathogenesis of MS, it is worth analyzing the disease from different points of view including different tissues. In this line, the role of CD4+ T cells was extensively studied considering both CSF and blood simultaneously. Transcriptomic analysis was performed on CSF-infiltrating CD4+ T cells from a cohort of 105 untreated patients with MS. Additionally, CSF was obtained from 11 controls and from 10 patients without myelin oligodendrocyte glycoprotein antibody-associated disease. Also, mononuclear cells were obtained from blood from four HC donors. In the literature were identified patients that showed different CSF-infiltrating CD4+ T cell responses against myelin and GDP-L-FS peptides. In 14 patients, mainly reactivity in IFN-γ and/or proliferation was observed against GDP-LFS peptides and these were classified as GDP-L-FS-responders. Additionally, 4 patients showed a response only to myelin basic protein (MBP) peptides (MBP responders) and 11 patients responded only against myelin oligodendrocyte glycoprotein (MOG), classified as MOG responders. Patients that did not show any reaction to autoantigen numbered 76 and were classified as non-responders. In a sub-cohort of patients with MS, an immunophenotyping of CSF-infiltrating and circulating lymphocytes was performed. Transcriptomic analysis was performed from effector memory CD27− and CD27+ CD4+ T cells sorted from the peripheral blood of 4 GDP-L-FS responders. The transcriptomic analysis revealed 265 DEGs among which 119 were upregulated and 146 downregulated. *ADGRG1*, *ADGRG5*, *CCL4*, *CCL5*, *CST7*, *CTSW*, *CX3CR1*, *ENC1*, *FCRL6*, *FGFBP2*, *GNLY*, *GZMB*, *GZMH*, *MYO6*, *PRF1*, *PRSS23*, *S1PR5*, *SLAMF7*, *SPON2*, *TGFBR3*, *TRGC2*, *ZEB2* were upregulated DEGs involved in CD4+ and CD8+ cytotoxicity. To the same purpose, DEGs encoding for transcription factors involved in the development of cytotoxic T cells were observed. Conversely, downregulated DEGs were linked to receptors for chemokines and cytokines. An association between T cell specificity and features of disease heterogeneity considering a T cell-mediated autoimmune disease was observed [48]. Another study was based on the blood or CSF of 79 MS patients and 38 controls without inflammatory disorders, with four different comparisons performed. In the 20 controls with both CSF and blood samples, 2556 upregulated and 2600 downregulated DEGs enriched for 61 GO terms were highlighted. On the other hand, the comparison of the 21 patients with both CSF and blood samples in MS showed 2102 upregulated and 2161 downregulated genes enriched for 176 GO terms. The two analyses shared 82% of DEGs mainly participating in migration and activation with “Movement of cell or subcellular component”, among the most enriched, in both comparisons. The upregulated DEGs in the CSF were associated with a Th1 phenotype. Also, MS and controls were compared after the CSF against blood comparison, which revealed *PASK* upregulated and *CYP51A1*, *LRRD1* and *YES1* downregulated. The last comparison was made among all MS against all the controls and highlighted 115 upregulated and 25 downregulated genes where most of the DEGs were enriched for mitochondrial functions [49]. Through multiple tissue studies, it is possible to analyze the function of sncRNAs as in a study of PBMCs, plasma, CSF cells and cell-free CSV from RRMS, SPMS, inflammatory neurological disease control (INDC) non-INDC. The cohort was made up of 12 patients in relapse and 11 patients in the remission phase for RRMS, 6 patients for SPMS, 11 non-INDC and 5 INDC. The fold change obtained by the DEGs of the PBMCs suggested that sncRNAs regulated the expression level of the transcriptome mainly through alternative splicing, RNA degradation and mRNA translation. Most of the sncRNAs were upregulated in RRMS over non-INDC in CSF and downregulated in PBMCs and plasma. Also, the identified miRNAs were associated with the pathways related to T and B cell activation, cytokine and chemokine signaling and transforming growth factor beta signaling [50]. Data about different tissues can also be retrieved in different databases. From the 3 RNA-seq dataset, all the data related to the keyword “multiple sclerosis” were retrieved. The databases used for the research were GEO, EBI-EMBL ArrayExpress and DDBJ Sequence Read Archive. All the data retrieved referred to human species. The datasets included in the work were obtained from seven different blood tissues (B cell, T cell, monocyte, platelets, neutrophils, natural killer cell and whole blood) and eight brain tissues (optic chiasm, corpus callosum, occipital cortex, astrocytes, frontal cortex, hippocampus, internal capsule and parietal cortex) from 207 MS patients and 348 controls. The number of lncRNAs was mainly associated with expression in blood rather than in the brain. A following WGCNA was made between the 5420 deregulated lncRNAs and 2051 DEGs that, after removing outlier samples, highlighted a network with 15 modules. From the analysis, the total number of lncRNAs resulting as upregulated was higher than the downregulated ones both in the brain and in blood. These results indicated that MS risk is related to an overexpression of lncRNA. Among the lncRNA found to be differentially expressed, some are more closely related to MS conditions. *NONHSAG081583.2* is an interesting lncRNA because it targets *CDH1* and *CDH2*, which encode for cadherin protein, the most abundant adhesion molecules that participate in nerve conduction in the synaptic junction. Another important highlighted lncRNA was *NONHSAG000840.2*, which targets *NOTCH2*. Some co-expressed protein coding genes are involved in immune response by leukocytes and interleukin. One important lncRNA resulting from that analysis is *NONHSAG049754.2*, which targets *TNFRSF10A*, a gene that encodes for the receptor of tumor necrosis factor cytokines, susceptible to developing MS. From the just mentioned analysis, it was seen that a key mechanism of lncRNA involved in MS is associated with the regulation of TNF cytokine receptors and ribonucleoprotein [51]. 

Multiple sclerosis was also included with other autoimmune diseases to study possible elements among them. Along with MS, the RNA sequencing data of multiple autoimmune diseases were retrieved about type 1 diabetes, systemic lupus erythematosus and rheumatoid arthritis for the identification of similar disease-specific signatures. The aim was the identification of key pathways that could be targeted for therapy. The study was performed on different tissues related to the pathology but it was limited because of the great difference between the samples and the age of the patients but also because the data were obtained by different teams. For MS, the tissue was the optic chiasm with five patients and five controls. The enrichment analysis of these disease-modified genes showed that all the diseases have several genes upregulated in the IFN I and II related pathways and antigen presentation, while the downregulation was associated with specific pathways; for example, lipid metabolism was enriched in MS. They also identified the risk genes of all the diseases using GWAS studies and found *TYK2*, *IKZF3*, *HLA-DQB1* and *HLA-DRB1*. The overlapping of the genes between two or more diseases is mostly related to inflammatory mediators and the signaling of IFNs. The similarities in the pathways were studied and translated in the identification of different drugs. Among them, JAK inhibitors that block IFN signaling were suggested; these classes of drugs were recently approved for the treatment of arthritis. Another suggested drug was a PI3K inhibitor, which had beneficial effects in animal models of MS, lupus and arthritis. However, PI3K inhibitors exacerbated inflammatory responses in the airways and gut. A gene associated to all the diseases was *TYK2*, a key component of the JAK-STAT signaling pathways. *TYK2* inhibitors are already in phase 3 clinical trials for psoriasis, an autoimmune disease [52]. 

In MS, the gender of patients also plays an important role in pathology and a multi-tissue study focused on the polymorphism rs755622 in the promoter region of the macrophage migration inhibitory factor. It is a modulator of the macrophages and microglia immune response and is associated with autoimmune diseases. Also, it has a homologous D-dopachrome tautomerase (DDT) that has a sex-specific disease modifier for MS because its high expression can promote MS progression in males but not in females. Using five large-scale eQTL and two RNA-seq datasets from the brain and blood to study the polymorphism, they found that the frequency of rs755622 and expression of DDT are significantly increased in males but not in females, suggesting that rs755622 influences the DDT expression level in males. In HC, the distribution of the SNP was not significantly different between genders [53]. 

Blood and the brain are not the only human tissues that show an imbalance in expression in MS. Indeed, spinal cord functions slowly deteriorate beyond age 40 in patients affected by MS in its progressive form. The genomic signatures of the brain and spinal cord were retrieved from the ARCHS4 library, which provided a high number of human RNA-seq data, to obtain two lists of genes specific for the signature. The focus of the work was the analysis of homeobox genes related to the ARCHS4 spinal cord-specific signatures. Starting from a list of 29 suitable homeobox genes, after analysis performed taking advantage of data obtained in the GTEx database, 10 genes were identified that, in physiological conditions, were expressed in the spinal cord and not, or poorly, in the CNS area. These genes resulted in overexpression in the spinal cord compared to the CNS. Among the HOX genes reported, only *HOXA5* encoded a protein that interacts with a member of the TGF-beta signaling pathway. The TGF-beta1 progliotic signature is characteristic of areas of incomplete demyelination. After the identification of the homeobox before-mentioned, a list of 14 transcription factors that had an interaction with them was retrieved. Among these transcription factor partners, *SMAD1* and *SOX2* belong to the astrocytosis-related co-expression module reported in the literature. *SMAD1* and *SOX2* had a progliotic effect considering the interaction with the androgen receptor (AR), GLIS3 (GLIS family zinc finger 3) and NFIB (nuclear factor I B). *SMAD1* is also involved in the *TGFB1* signaling pathways. The involvement in spinal cord gliosis linked *HOXA5* to *SOX1* and *SMAD2*. Research in the JASPAR library confirmed that a list of predicted targets of *HOXA5* includes *SMAD1*, *SMAD3*, *TGFB1* and *TGFBR2*. Data mining analysis suggested that AR has a negative regulation effect on the progliotic pathway associated with spinal cord-overexpressed HOX proteins. The AR anti-inflammatory pathway promotes myelin repair and compensates for the progliotic effect of *TGFB1*. In adults beyond 40 years old, the physiological decline in AR ligands leads to an imbalance between AR and the TGFB1 pathway, a crucial event for the gliosis and alteration of myelin homeostasis that could translate into myelin degeneration [54]. 

The comparison of different cells and tissues was possible thanks to transcriptomics and allowed the discovery of specific patterns of gene expression. Studies based on multiple tissues allowed the analysis of MS from different points of view simultaneously. From the analysis of both CD4+ T cells and CSF it was possible to identify DEGS encoding for transcription factors involved in cytotoxic T cells and in addition CSF compared to blood revealed different interesting DEGs. Also, the role of ncRNA was clarified in these studies, in particular the involvement of different miRNAs in many pathways and the role of lncRNA in the synaptic junction, NOTCH pathway and tumor necrosis factor pathway. Paying attention to optic chiasm, it was also possible to point out several genes upregulated in the IFN I and II related pathways and antigen presentation.

An overview the studies exploring transcriptomes in multiple tissues is reported in Table 5.

#### 3.1.6. Other Tissues

The correlation between MS and smoke was inspected through the changes in the methylomic and transcriptomic profiles that occurred in cells obtained with the bronchoalveolar lavage (BAL) of patients with MS both against healthy individuals and against smoking patients. Only females were included in the study: 9 smoker MS patients, 8 non-smoker MS patients, 10 smoker controls and 12 non-smoker controls. The analysis revealed 1376 bisulfite (BS), 131 DNA methylation (5mC) and 4 DNA hydroxymethylation (5hmC) positions differently methylated in the comparison of MS smoking or not while 1821 BS-DMPs, 234 5mC-DMPs and 1 5hmC positions were found in controls. Even if the controls had higher changes, most of the alterations were the same between controls and MS. The analysis revealed that the changes highlighted in BAL were significantly higher than the ones observed in blood. The GO analysis was conducted over 827 genes in MS and 1036 genes in the control groups. The enrichment showed most processes related to cancer. Minimum differences were observed between the two groups and were associated with neurological diseases, cell survival, cell death, gene expression in MS, lipid metabolism and cell cycle in controls. On the other hand, the transcriptomic analysis revealed no DEGs between MS and controls. Thus, the authors concluded that the machinery of transcription and translation resulted in reduced MS while the cellular motility was enhanced with slight differences [55]. Even the extracellular vesicles of plasma could clarify the aspects of pathology using circRNAs. In a study, the role of the transcriptomic profile in the extracellular vesicles extracted from plasma was inspected. From a cohort of 10 RRMS, 10 SPMS and 8 healthy individuals with correspondence in age and sex, the authors put, for the first time, a focus on circRNAs. The interesting results obtained from the authors suggested that circRNAs were the most expressed non-coding RNAs in the vesicles and the distributions for RRMS, SPMS and controls were comparable. Particularly, SPMS and RRMS shared 1939 circRNAs (36.4%), 2734 were exclusively present in RRMS, while 658 only in SPMS. Noteworthily, SPMS expressed a lower number of circRNAs. Then, they performed the same inspection on leukocytes. Thus, from a comparison of the profile of non-coding RNAs observed in vesicles against the one in the cytoplasm of leukocytes, there was no linear correspondence, not even for circRNAs. Also, the circRNAs in vesicles were smaller and more poorly structured than the leukocyte ones [56]. 

Transcriptomic analysis makes possible the study of different kinds of tissue; from a study based on BAL it was possible to observe that MS shows reduced machinery of transcription and translation. It was also observed that in extracellular vesicles of plasma the circRNAs were the most expressed non-coding RNAs and, in vesicles, this kind of RNA is smaller and more poorly structured than in leukocytes.

An overview the studies exploring transcriptomes in other tissues is reported in Table 6.

Along with classical RNA-seq analysis where bulk is used to perform transcriptomic inspection, single-cell studies are becoming useful for obtaining gene expression information with higher resolution. Indeed, with single-cell inspection it is possible put a focus on a specific cell type, removing the noise from analysis. Multiple sclerosis is a complex highly heterogenous disease, and attention to a specific kind of cell, such as microglia, astrocyte or oligodendrocyte progenitor cells, can increase the possibility of revealing the key point of the disease [57].

Lindeman et al. used the single-cell RNA-sequencing (scRNA-seq) of the B cells obtained from the CSF samples of MS patients for studying the B cell response in MS. They analyzed the transcriptomic profiles of 1621 antibody-secreting cells (ASCs) from 21 MS patients. Using cytometry, only CD38+, CD27+ and CD19+ single ASCs were sorted. With the sequences obtained from scRNA-seq, using the BraCeR pipeline they obtained the full length of the B cell receptors. As expected, CD27+ memory B cells dominated among sorted CD19+ B cells. IgG1 was by far the most prevalent isotype among ASCs. They confirmed that the *IGHV4* gene is dominant in the CSF and brain of patients with MS and CIS. The results revealed a common pattern across patients with a preferential VH:VL linked to the G1m1 allotype in ASCs. The G1m1 allotype dominates the intrathecal humoral immune response in G1m1/G1m3 heterozygous MS patients. They observed extensive connections between the intrathecal memory and ASC compartments and found evidence of isotype switching from IgM to IgG1, from IgG1 to IgA1 and from IgG1 to IgG2. The pairing of *IGHV4* and *IGKV1* in the BCR of ASCs expressing G1m1 bears a resemblance to stereotyped B cell responses observed in other diseases that are driven by particular antigenic epitopes. Nevertheless, one main limitation of the study is related to the lack of a control group of patients with other neurological diseases [58]. Ramesh et Al. performed a scRNA-seq on paired CSF and blood from HC, patients with RRMS and subjects with other neurologic diseases. Moreover, on a subset of these subjects, single-cell immunoglobulin sequencing was performed. Further, paired CSF and blood B cell subsets (on seven patients) were isolated using fluorescence-activated cell sorting for bulk RNA-seq. The experiments demonstrated that NF-κB and cholesterol biosynthesis pathways were activated. Also, specific cytokine and chemokine receptors were upregulated in CSF memory B cells. In addition, they assessed the presence of non-host and human endogenous retrovirus (ERV) transcripts to search for evidence of viral transcription in both blood and CSF B cells. No ERVs/repetitive elements were observed between individual blood and CSF B cell subsets. SMAD/TGF-β1 signaling was downregulated in CSF plasmablasts/plasma cells. Clonally expanded, somatically hypermutated IgM+ and IgG1+ CSF B cells were associated with inflammation, blood–brain barrier breakdown and intrathecal Ig synthesis. Their findings supported the hypothesis that, in MS, CSF B cells were driven to an inflammatory and clonally expanded memory and plasmablast/plasma cell phenotype [59]. Schafflick et al. used a microfluidics-based scRNAseq to obtain transcriptome data related to blood and CSF. Using an analytical approach, named cell set enrichment analysis, they reconstructed the leukocyte lineages of the starting tissue. This analysis classified the cells using the DEGs. In this procedure, the cells were first ordered by a transcriptional phenotype of interest. The cell sets enriched in MS when compared to controls expressed signatures of Th1 and T follicular helper (TFH) cells. The TFH signature was enriched in the CSF but not in the blood. These cluster cells can cause the known expansion of B lineage cells in the CSF in MS. From the analysis of blood cells it resulted that there are no significant differences in composition between MS and control, as was confirmed by flow cytometry. Focusing on transcriptional changes related to blood cells, it was observed that the altered processes were involved in the induction of activation markers, specific cytokine receptors and trafficking molecules in T cells. Conversely, the cell type composition in CSF was altered in the comparison. The study of CSF also showed an enrichment of myeloid dendritic cells and Tregs as well as an expansion of cytotoxic phenotype CD4+ T cells that could be involved in local MS pathology. Myeloid dendritic cells 1 in the CSF expressed markers of cross-presenting capacity such as *XCR1* and *WDFY*4 while NK2 cells in the CSF expressed the corresponding ligands *XCL1* and *XCL2* indicating that cell types equipped for cross-presentation and antiviral defense circulate the CSF. All the results reported provided evidence for compartmentalized mechanisms driving human autoimmunity in the brain. Also, from the analysis it resulted that TFH cells enhanced B cell enrichment in the CNS in EAE. The interaction between TFH cells and B cells in CSF could drive CNS autoimmune reactions. They also found a cluster expansion of CD4+ T cells with the cytotoxic phenotype in MS and the marker *EOMES,* already known as a genetic risk locus for RRMS [60]. Another study aimed to compare gene expression patterns of CSF cells from MS-discordant monozygotic twin pairs. Some patients showed subclinical neuroinflammation (SCNI) with small MRI lesions. Some other subjects had OCBs. The patients selected were monozygotic twins where one sibling was clinically definite as MS and the other was clinically “healthy” with an MRI and/or immunological evidence for SCNI. scRNA-seq was used to identify clonally expanded CD8+ T cells, plasmablasts and, to a lesser extent, CD4+ T cells both from MS patients and from subjects with SCNI. They found that clonal expansions of CD8+ T cells, B cells and CD4+ T cells were conspicuous even in the prodromal stage of MS in familiarly predisposed subjects, in particular in the CD8+ T cell compartment and these cells showed characteristics of activated tissue-resident memory T (TRM) cells. These characteristics are more present in MS subjects and lower in subjects with SCNI. Their findings demonstrated that even the earliest stage of MS was characterized by a synergistic activation of the main components of the adaptive immune system with a striking contribution of recently activated, clonally expanded CD8+ T cells with a TRM phenotype [61]. In the study of Kaufmann et al., the immune cell colonization of the central nervous system in MS was inspected. Indeed, along with their accumulation behind the blood-brain barrier, they can lead to the therapeutic resistance of PMS. They tracked CNS-homing immune cells in the peripheral blood of 31 MS patients and 31 matched healthy individuals in an integrated analysis of 497.705 single-cell transcriptomes and 355.433 surface protein profiles from 71 samples. Through spatial RNA sequencing, they localized the cells in the post mortem brain tissue of six PMS patients against four control brains (20 samples, 85.000 spot transcriptomes). To perform the study, the PBMCs of RRMS patients treated with or without natalizumab were sampled and underwent scRNA-seq and surface marker profiling. The authors identified a specific pathogenic CD161+/lymphotoxin beta (LTB)+ T cell population that resided in the brains of PMS and that could begin earlier in the disease course patients. Their data suggested that this population could mobilize to the blood using the integrin-blocking antibody natalizumab in RRMS patients [62]. To investigate the role of Zinc finger E-box-binding homeobox (ZEB1) by promoting Th1 and Th17 differentiation, Qian et al. used scRNA-seq to evaluate the transcriptomic profiles of human CD4+ T cells in MS. The transcriptomic analysis of PBMCs isolated from the blood of 25 RRMS patients showed 87 genes associated to MS significantly enriched and able to affect both CD4+ T cells and Th1- and Th17-related pathways. Among highly transcribed genes, *ZEB1*, *IL-2RA* and *ALPK2*, both in Th17 and Th1 cells, were identified. The authors investigated *ZEB1* for its important role in CD4+ T cell biology. Noteworthily, an upregulation of *ZEB1*, *IL-17* and IFN-γ in the myelin-reactive Th17 cells of MS patients compared to control patients was shown. ZEB was fundamental for Th1 and Th17 differentiation in humans and mice. For studying *ZEB1,* they used EAE models. *ZEB1* loss in T cells functionally ameliorates the development and the severity of EAE in a mouse model of MS. Furthermore, the involvement of *ZEB1* in JAK-STAT signaling was reported. Indeed, *ZEB1* inhibits miR-101-3, which represses *JAK2* expression and STAT3/STAT4 phosphorylation, leading to the reduction of *IL-17* and IFN-γ as well as the reduction of the inflammation in MS. Overall, the study showed a mechanism whereby, through the pathway mediated by *ZEB1*, miR-101-3p and *JAK2* could be a potential therapeutic target to avoid pathogenic differentiation in MS patients [63]. Afshin Derakhshani et al. used the raw data of scRNA-seq in patients with MS. They used the Scanpy package version 1.7 to re-analyze the scRNA-seq data and showed that Cytotoxic T-lymphocyte antigen-4 (CTLA-4) and programmed death-ligand 1 (PD-L1) were expressed mainly on the naïve T cells, regulatory T cells and activated CD8+ T cells. Through Real-Time PCR, they showed a change in expression levels of the *CTLA-4* and *PD-L1* genes after treatment with Fingolimod, IFNβ-1α, Glatiramer Acetate and Dimethyl Fumarate. *CTLA-4* and *PD-L1*, as inhibitory immune checkpoints, participated in the development of MS. The expression of *CTLA-4* and *PD-L1* decreased in MS patients compared to healthy people. This study showed, especially with the Fingolimod treatment, the increased expression of *CTLA-4* and *PD-L1* in MS patients compared to untreated MS patients [64]. Kihara et al. used snRNA-seq to generate a high-quality RRMS vs. SPMS comparison to try to shed light on the implications for the efficacy of Fingolimod. The tissues used for the comparison were prefrontal cortices taken from five RRMS patients and five SPMS patients that did not show any detectable demyelination. From the comparison of the gene expression profiles of RRMS and SPMS excitatory neurons resulted 1851 DEGs. Among the genes upregulated in RRMS, immune response pathways were highlighted as enriched pathways from the analysis performed on Reactome. The markers for excitatory neurons were significantly downregulated in RRMS compared to SPMS. The most identified cells in both RRMS and SPMS samples were oligodendrocytes (Ols). The analysis of this cell type showed 1145 DEGs in the comparison of RRMS vs. SPMS and, among these DEGS, around 94% were upregulated in RRMS. These results suggested that the maturation and myelination of oligodendrocyte progenitor cells (OPCs) was more active in the RRMS brain than in the SPMS one. The RRMS astrocytes expressed some genes related to antioxidant pathways (*SLC7A11*, *ME1*, *FTH1*) compared to the SPMS astrocytes. These results were a confirmation that SPMS astrocytes reacted to the severe oxidative stress that inhibits OPC maturation. In addition, genes involved in de novo sphingolipid synthesis were downregulated in the Ols and OPCs of SPMS compared to RRMS. This result confirmed a dysfunction in myelin synthesis consequential to MS progression. Furthermore, astrocytic sphingosine kinases (SPHK1/2), the enzymes required to activate and phosphorylate the fingolimod, were downregulated in the SPMS brain. In the study, an animal model analysis was integrated. There, it was reported how Sphk1/2 null mice lost fingolimod activity. The reduction in the expression of protein kinase C, responsible for the induction of SPHK1 expression in SPMS astrocytes, could explain the relative downregulation of *SPHK1* [65]. Sunjay Jude Fernandes et al. deep-characterized paired chromatins and transcriptomes in four immune cell types from RRMS patients. Through transposase-accessible chromatin (ATAC-Seq), they identified differentially accessible regions between RRMS patients and HC, primarily in CD4+ and CD19+. In particular, they showed the increased potential for transcriptional activity in CD4+ in RRMS close to differentially accessible regions of CD4+ cells; RNA-Seq showed 42 DEGs. In this work, they combined the paired ATAC-Seq and RNA-Seq data and identified *CCDC114* as a novel candidate for study in MS and *SERTAD1* as dysregulated in MS [66]. Liu et al. developed a Bayesian framework modified Integrative Risk Gene Selector capable of incorporating and summarizing the genetic information and characteristics of the largest current GWAS MS. The aim was the identification of priority genes (MS-PRGenes) associated with the disease. Additionally, MS-PRGenes were validated in 19 tissues by a two-sample Mendelian randomization (2SMR) approach. Single-cell enrichment analyses were conducted to better understand the function of MS-PRGenes and investigated potential MS drug strategies. The authors used 200 SNPs reported in the GWAS of 14.802 MS cases and 26.703 control subjects. Based on the results obtained, 163 MS-PRGenes from 200 genetic loci reported in the current MS GWAS were identified. Additionally, 35 MS-PRGenes were validated through the 2SMR approach. The results showed the presence of MS-PRGenes with significantly higher pLi scores and high overlap rates compared to known genes associated with the risk of disease. Among these genes were included *KMT2A*, *LITAF*, *CD48*, *CD86*, *CD9*, *MAF*, *MYC, TNFAIP3*, *LPIN1*, *ATXN1*, *JARID2*, *SATB1*, *GATA3*, *ELMO1*, *SGK1* and *ZNF217*. In addition, single-cell enrichment analysis demonstrated that MS-PRGenes were more enriched in cells’ related immune function, such as macrophages and microglia, thereby signaling disease-associated inflammatory signaling pathways. Thus, the study predicted and confirmed a set of genes associated with MS risk, thus proposing MS-PRGenes as potential therapeutic targets for the disease [67]. Single-cell transcriptomics in combination with analytical techniques allow the characterization of the specifics of immune cells. A study performed using single-cell analysis revealed that in the CSF of MS patients there is an enrichment of the TFH signature. Another study identified a specific pathogenic CD161+/lymphotoxin beta (LTB)+ T cell population that resided in the brains of MS patients and that could begin earlier in the disease. Using single-cells analysis for MS diseases, the possibility to study the change of immune cells in the creation of the specific antibody and receptor is improved. The scRNA-seq of the B cells obtained from CSF and the BraCeR pipeline shows the full length of B cell receptors. Single cells can also give us information about how specific types of cells respond to treatment. Fingolimod treatment increased the expression of CTLA-4 and PD-L1 in the naïve T cells, regulatory T cells and activated CD8+ T cells of MS patients. In the SPMS brain, and not in the RPMS brain, there is a downregulation of astrocytic sphingosine kinases (SPHK1/2) fundamental for the activation of fingolimod. 

An overview the studies exploring transcriptomes in single-cells is reported in Table 7.

## 4. Conclusions

Transcriptomic studies evidenced the intricate gene network involved in MS. Next-Generation Sequencing big data were able to improve the quality of the analysis. Interestingly, the landscape of the transcriptome identified that RRMS and SPMS stages express different signatures at the level of neuron repair pathways modulating in opposite direction specific miRNAs. Along with miRNAs, lncRNAs and circRNAs were found as new key elements deregulated in MS. Men and women with MS presented different transcriptomic profiles before and after drug therapy with IFN-β1. With an effect comparable to fingolimod, the transcriptome showed different biological processes specific to the two drugs. Demyelination was associated with the activation of the neddylation pathway and the inflammatory profile was linked to neuron degeneration areas with spatial transcriptomics. Furthermore, a main focus on single-cell studies allowed the identification of clusters of immune cells with very high resolution to study in detail their interaction. In this line, the reported literature highlights how much the use of RNA-seq in the study of complex pathologies, such as MS, is a valid strategy to shed light on disrupted mechanisms.

## Figures and Tables

**Table 1:** Overview of the studies exploring transcriptomes in human PBMCs.

MS Cohort	Disease State	Mean Age	Gender	Source	Methodology	Treatment	RNA Type	Main Conclusions	Reference
9	RRMS = 3	45	2F, 1M	Leukocytes	Comparative transcriptomics	1 Avonex, 1 IFN-β, 1 UT	Total RNA	Inflammatory profile is activated in RRMS patients, while neuronal repair is decreased in SPMS	[21]
	SPMS = 6	48.5	4F, 2M			6 natalizumab			
106	CIS = 40 RRMS = 66	34.5 (from 19 to 58)	74F, 35M	CD4+ T cells, CD8+ T cells, CD14+ monocytes	Comparative transcriptomics	UT	Total RNA	Demyelination and inflammatory cell infiltration are linked to the activation of the neddylation pathway	[22]
4	RRMS = 4	37.8 ± 7.3	4F	CD4+ T cells	Comparative transcriptomics	UT	Total RNA	The identified DEGs are linked to a hormone-dependent transcriptional activation	[23]
22	RRMS = 12	N.P.	6F, 6M	CD4+ T cells, CD8+ T cells	Comparative transcriptomics	UT	Total RNA	The most enriched biological process terms are the same for CD4+ and CD8+ T cells and are linked to mRNA processing and translation	[24]
	SPMS = 10	N.P.	7F, 3M						
30	RRMS = 30	36.0 ± 11.0	21F, 9M	PBMCs	Comparative transcriptomics	11 IFN-β1b, 19 IFNb1a	Total RNA	Therapy with IFN-ꞵ1 is sex-dependent	[25]
24	RRMS = 24	27.9 ± 8.4	16F, 8M	CD14+ T cells	Comparative transcriptomics	UT 3 months, then Fingolimod	Total RNA	Fingolimod modulates myeloid cells thorough Wnt pathway	[26]
100	RRMS = 55	36.9 ± 8.3	7F, 2M	NK cells	Comparative transcriptomics	UT	Total RNA	Fingolimod and IFN-ꞵ1 alter the transcriptomic profiles in different manners through cell cycle, protein biogenesis, mitochondrial processes, RNA metabolism and intracellular signal transduction pathway, regulation of receptor activity, the cytoplasmic sequestration of transcription factors, protein modification, complex formation, disassembly and localization, oxidation-reduction processes or neuroactive ligand–receptor interactions, ferroptosis, phagosomes and tRNA synthesis	[27]
		34.7 ± 11.2	26F, 10M			IFN-β1			
		32.08	35F, 20M			Fingolimod			
5	RRMS = 5	N.P.	N.P.	CD4 T cells, CD8 T cells, monocytes, B cells	Comparative transcriptomics	2 IFN-β, 1 Glatirameracetate, 1 natalizumab, 1 UT	Total RNA	Vitamin D exposure in MS changes activity of genes involved in its processing such as *NR4A2*, *NR4A3*, *CD14* and *VDR*	[28]
50	RRMS = 50	36.1 ± 8.3	6F, 4M	CD4 T cells, NK cells, Helper T cells, B cells	Comparative transcriptomics	10 UT	Total RNA	Responders and non-responders of the fingolimod treatment differ in changing of the lymphocyte profile, moving from an anti-inflammatory to an antioxidant context	[29]
		40.6 ± 9.7	25F, 15M			18 natalizumab, 22 not natalizumab			
24	SPMS = 12	60.2 ± 8.3	9F, 3M	CD4+ T cells	Comparative transcriptomics	UT	miRNAs	Expression of some miRNAs in SPMS contrast with RRMS so SPMS is a non-inflammatory mediated disease	[30]
	SPMS = 12	61.4 ± 6.5	8F, 4M						
101	RRMS = 19	37.9	16F, 3M	Exosome by serum	Comparative transcriptomics	Methylprednisolone	miRNAs	Expression of miRNA in serum correlates with lesions in magnetic resonance imaging	[31]
	RRMS = 63	36.4	46F, 17M						
	RRMS = 19	34.4	16F, 3M						
8	RIS = 8	28.6 ± 6.8	4F, 4M	PBMCs	Comparative transcriptomics	N.P.	Total RNA	Transcriptome proved the dysregulation of pathways mediated by cytokines and chemokines. Radiologically Isolated Syndrome could the preclinical stage and/or subclinical form of MS	[33]

N.P. stands for not provided. In gender column, F stands for female, M for male. In treatment column, UT stands for untreated.

**Table 2:** Overview of the studies exploring transcriptomes in the human CSF.

MS Cohort	Disease State	Mean Age	Gender	Source	Methodology	Treatment	RNA Type	Main Conclusions	Reference
4	RRMS = 4	31.3	1F, 3M	B cells	ImmunoSEQ	1 dimethylfumarate	mRNA	B cell response in MS is heterogeneous and partly directed against intracellular autoantigens released during tissue destruction	[34]
10	RRMS = 10	37.2	9F, 1M	B cells	ImmunoSEQ	2 IFN-β1, 1 Glitaramer acetate, 7 UT	mRNA	Transcriptomes were dominated by a few B cell clones. B cells may mature on both sides of the blood–CSF barrier. IgG-producing CSF B cells in MS patients more frequently used IGHV4 genes with a high number of replacement mutations	[35]
2	RRMS = 2	31	2F	B cells	ImmunoSEQ	Natalizumab, IFN-β1α	mRNA	Persistence of intrathecal oligoclonal B cells	[36]

In gender column, F stands for female, M for male. In treatment column, UT stands for untreated.

**Table 3:** Overview of the studies exploring transcriptomes in the human blood.

MS Cohort	Disease State	Mean Age	Gender	Source	Methodology	Treatment	RNA Type	Main Conclusions	Reference
7	RRMS = 7	30.8 ± 7	5F. 2M	Whole blood	Comparative transcriptomics	2 IFN-β1α, 4 Fingolimod, 1 UT	Total RNA	MS transcriptome changes in a time-dependent manner	[38]
51	N.P.	46.2 ± 7.5	38F, 13M	Whole blood	sQTL	N.P.	Total RNA	SNPs support alternative splicing	[39]
51	N.P.	46.2 ± 7.5	38F, 13M	Whole blood	eQTL	N.P.	lncRNAs	Many lncRNAs expressed in cerebral and closer tissues carried SNPs responsible for alteration of secondary structure of lncRNAs	[40]
19	RRMS = 19	15.5 ± 2.7	10F, 9M	Peripheral blood	Comparative transcriptomics	9 DMT IFN-β1α, 10 DMT	miRNAs, mRNAs	Enriched KEGG pathways are Prion disease, fatty acid biosynthesis, cell cycles, Hippo signaling pathway and adherence junction	[41]
120	RRMS = 20 SPMS = 10	35.7	23F, 7M	Whole blood	Comparative transcriptomics	N.P.	circRNAs	Six circRNAs were deregulated in a sex-dependent manner	[42]
	RRMS = 62 SPMS = 8	41.1	14F, 6M						
	RRMS = 20 SPMS = 20	39.3	24F, 16M						

N.P. stands for not provided. In gender column, F stands for female, M for male. In treatment column, UT stands for untreated.

**Table 4:** Overview of the studies exploring transcriptomes in the human brain.

MS Cohort	Disease State	Mean Age	Gender	Source	Methodology	Treatment	RNA Type	Main Conclusions	Reference
10	PMS = 10	52.4	5F, 5M	Normal appearing white matter, active, chronic active, inactive, remyelinating	Database	N.P.	Total RNA	The database collects the transcriptomic profiles of 98 human brains obtained in autopsy	[43]
5	SPMS = 1 PMS = 3 N.P. = 1	57.6	5F	Corpus callosum, hippocampus, internal capsule, optic chiasm, frontal and parietal cortex	Comparative transcriptomics	4 UT, 1 N.P.	Total RNA	The heat shock protein family were deregulated in six different brain areas	[44]
10	PMS = 10	52.4	5F, 5M	Chronic active, remyelinating, normal appearing white matter	Machine learning	N.P.	Total RNA	Higher abundance of M2 in the lesions of MS during the inactive phase	[45]
13	SPMS = 13	48.6 (from original cohort of 15 SPMS)	11F, 3M (from original cohort of 15 SPMS)	Cortical brains	Spatial transcriptomics	1 IFN-β, 1 azathioprine, 1 alemtuzumab, 1 N.P.	mRNAs	Areas with neuron degeneration are connected with inflammatory profile	[47]

N.P. stands for not provided. In gender column, F stands for female, M for male. In treatment column, UT stands for untreated.

**Table 5:** Overview of the studies exploring transcriptomes in multiple tissues.

MS Cohort	Disease State	Mean Age	Gender	Source	Methodology	Treatment	RNA Type	Main Conclusions	Reference
105	RRMS = 82	35.3 ± 9.0	F/M = 1.9	CD4+ T cells, CSF	Comparative transcriptomic	UT	Total RNA	Identified DEGs encoding for transcription factors involved in cytotoxic T cells	[48]
	PMS = 9	46.9 ± 8.0	F/M = 2						
	RIS/CIS = 14	30.6 ± 10.3	F/M = 1.8						
79	RRMS = N.P., N.P.	44 ± 13.1	26F, 15M	CD4+ T cells, CSF	Comparative transcriptomic	UT	Total RNA	Cerebrospinal fluid comparison showed differences associated with migration and activation of “Movement of cell or subcellular component”; CSF against blood revealed upregulation of *PASK* and downregulation of *CYP51A1*, *LRRD1* and *YES1*; MS and control differ for mitochondrial functions	[49]
29	RRMS (remission) = 11	37	11F	PBMCs, plasma, CSF cells and cell-free CSV	Comparative transcriptomic	1 IFN-β1α	sncRNAs	miRNAs are involved in pathways related to T and B cell activation, cytokine and chemokine signaling and transforming growth factor beta signaling	[50]
	RRMS (relapse) = 12	43	10F, 2M			2 methylprednisolone			
	SPMS = 5 (1 repeated)	56	3F, 2M			1 methylprednisolone			
207	RRMS = N.P., N.P.	N.P.	N.P.	B cell, T cell, monocyte, platelets, neutrophils, natural killer cell, whole blood, optic chiasm, corpus callosum, occipital cortex, astrocytes, frontal cortex, hippocampus, internal capsule, parietal cortex	Comparative transcriptomic, WGCNA	N.P.	lncRNAs	*NONHSAG081583.2* could play a role in synaptic junction, *NONHSAG000840.2* in NOTCH pathway; *NONHSAG049754.2* in tumor necrosis factor pathway	[51]
5	SPMS = N.P., PMS = N.P.	56.2	5F	Optic chiasm	Comparative transcriptomic	UT	mRNAs	Common to four immune diseases, several genes upregulated in the IFN I- and II-related pathways and antigen presentation; downregulation is disease-specific pathways; lipid metabolism was enriched in MS	[52]
11	RRMS = 5 N.P. = 6	N.P.	N.P.	Optic chiasm, blood	Comparative transcriptomic, eQTL	UT	Total RNA	D-dopachrome tautomerase expression due to rs755622 SNP is sex dependent	[53]

**Table 6:** Overview of the studies exploring transcriptomes in other tissues.

MS Cohort	Disease State	Mean Age	Gender	Source	Methodology	Treatment	RNA Type	Main Conclusions	Reference
17	nine smokers	38.0	17F	BAL	Comparative transcriptomic	UT	Total RNA	Multiple sclerosis shows reduced machinery of transcription and translation and enhanced cellular motility	[55]
	eight non-smokers	43.0							
20	RRMS = 10	39.7 ± 13.7	5M	Extracellular vesicles of plasma	Comparative transcriptomic	UT	circRNAs	circRNAs are the most expressed non-coding RNAs and in vesicles are smaller and more poorly structured than in leukocytes	[56]
		42.0 ± 19.8	5F						
	SPMS = 10	54.8 ± 6.2	5M						
		48.7 ± 6.7	5F						

In gender column, F stands for female, M for male. In treatment column, UT stands for untreated. 4.7 Single-cell studies.

**Table 7:** Overview of the studies exploring transcriptomes in single-cells.

MS Cohort	Disease State	Mean Age	Gender	Source	Methodology	Treatment	RNA Type	Main Conclusions	Reference
21	RRMS = 20	35	13F, 8M	CSF	Comparative transcriptomics	2 methylprednisolone, 19 UT	mRNA	Strong connection between a G1m allotype and the heavy- and light-chain variable gene in MS patients	[58]
	SPMS = 1								
18	RRMS = 16	22–54	15F, 5M	CSF (lymphocytes), Blood (PBMCs)	Comparative transcriptomics	UT	Total RNA or mRNA	NF-κB and cholesterol biosynthesis pathways were activated and specific cytokine and chemokine receptors were upregulated in CSF memory B cells. In MS, CSF B cells are driven to an inflammatory and clonally expanded memory and plasmablast/plasma cell phenotype	[59]
	CIS = 2	35–53							
39	RRMS = 39	33.7 ± 9.5	30F, 9M	CSF, Blood	Comparative transcriptomics	UT	mRNA	Evidence of autoimmunity in the brain. From the analysis it resulted also that TFH cells enhanced B cell enrichment in the CNS in EAE. The interaction between TFH cells and B cells in CSF may drive CNS autoimmune reactions	[60]
12	RRMS = 3	35.3 ± 5.9	3F	CSF	Comparative transcriptomics	1 IFN-β, 1 Natalizumab, 1 Teriflunomide	Total RNA	Earliest stage of MS is characterized by the activation of the main components of the adaptive immune system, with a striking contribution of recently activated, clonally expanded CD8+ T cells with a TRM phenotype	[61]
	SPMS = 1	50	1F			1 Intrathecal steroids			
31	RRMS = 21	36.9	5F, 5M	PBMC	Spatial transcriptomics	10 Natalizumab	mRNA	Identification of a specific population of CD161 + /lymphotoxin beta (LTB)+ T cell in the CNS. This may begin earlier in the disease course and they can be mobilized to the blood by usage of natalizumab in relapsing-remitting MS patients	[62]
		31	11F			11 UT			
	PPMS = 10	48.4	3F, 7M			10 UT			
25	RRMS = 25	N.P.	N.P.	PBMCs	Comparative transcriptomics	N.P.	mRNA	Zeb is fundamental for the differentiation of Th1 and Th17 differentiation. The ZEB1-JAK2 axis may possibly serve as target for MS treatment	[63]
45	RRMS = 45	From 20 to 59	32F, 13M	PBMCs	Comparative transcriptomics	Fingolimod, IFNβ-1α, Glatiramer Acetate, and dimethylfumarate	N.P.	Especially with the Fingolimod treatment, increased gene expression of CTLA-4 and PD-L1 in affected people compared to untreated affected patients	[64]
10	RRMS = 5	51.8 ± 6.5	2F, 3M	Prefrontal cortices	Comparative transcriptomics	2 Avonex, 1 Avonex+methylprednisolone,2 UT	Nucleus RNA	Difference in expression of S1PR genes and genes related to de novo sphingolipid synthesis may explain the difference in efficacy of fingolimod among RRMS and SPMS patients	[65]
	SPMS = 5	57.0 ± 6.6	3F, 2M			1 Methylprednisolone, 1 Avonex+copaxone, 3 UT			
4	RRMS = 4	N.P.	4F	CD4, CD8, CD14 e CD19	Comparative transcriptomics	N.P.	mRNA	New insight into the primary role of CD4+ and CD19+ cells in MS	[66]
4	SPMS = 3	50.0 ± 3.1	1F, 2M	White matter regions	Comparative transcriptomics	UT	Nucleic RNA (Single-cell)	Obtainment of a high-confidence MS risk gene set that integrates genomic, epigenomic, transcriptomic, single-cell and drug data	[67]
	PPMS = 1	37.0	1M						
10	N.P.	N.P.	N.P.	Microglia from corpus callosum			Total RNA (bulk cell)		

N.P. stands for not provided. In gender column, F stands for female, M for male. In treatment column, UT stands for untreated.

## Data Availability

Not applicable.

## Abbreviations

MS	Multiple sclerosis
CNS	central nervous system
CIS	clinically isolated syndrome
RRMS	relapsing remitting MS
SPMS	secondary progressive MS
PPMS	primary progressive MS
Th	T helper
IFN	interferon
IFN-β	interferon-β
HTS	high-throughput sequencing
NGS	Next-Generation Sequencing
lncRNAs	long non-coding RNAs
sncRNAs	small non-coding RNAs
circRNAs	circular RNAs
eQTL	expression Quantitative Trait Loci
sQTL	cis-splicing Quantitative Trait Loci
SNPs	single nucleotide polymorphisms
GEO	Gene Expression Omnibus
CSF	cerebrospinal fluid
PBMCs	peripheral blood mononuclear cells
DEGs	differentially expressed genes
GSEA	gene set enrichment analysis
GO	Gene Ontology
KEGG	Kyoto Encyclopedia of Genes and Genomes
HC	healthy controls
EAE	experimental autoimmune encephalomyelitis
RIS	Radiologically Isolated Syndrome
IGHV	heavy chain immunoglobulin gene
OCBs	oligoclonal bands
rOCB	recombinant OCB
WGCNA	weighted gene coexpression network analysis
INDC	inflammatory neurological disease control
DDT	D-dopachrome tautomerase
BAL	bronchoalveolar lavage
BS	bisulfite
5mC	DNA methylation
5hmC	DNA hydroxymethylation
scRNA-seq	single-cell RNA-sequencing
ERV	endogenous retrovirus
TFH	T follicular helper
SCNI	subclinical neuroinflammation
Ols	oligodendrocytes
OPCs	oligodendrocyte progenitor cells
2SMR	Mendelian randomization
